# Modafinil Induces Rapid-Onset Behavioral Sensitization and Cross-Sensitization with Cocaine in Mice: Implications for the Addictive Potential of Modafinil

**DOI:** 10.3389/fphar.2016.00420

**Published:** 2016-11-07

**Authors:** Raphael Wuo-Silva, Daniela F. Fukushiro, André W. Hollais, Renan Santos-Baldaia, Elisa Mári-Kawamoto, Laís F. Berro, Thaís S. Yokoyama, Leonardo B. Lopes-Silva, Carolina S. Bizerra, Roberta Procópio-Souza, Debora Hashiguchi, Lilian A. Figueiredo, Jose L. Costa, Roberto Frussa-Filho, Beatriz M. Longo

**Affiliations:** ^1^Laboratory of Neurophysiology, Department of Physiology, Universidade Federal de São PauloSão Paulo, Brazil; ^2^Department of Pharmacology, Universidade Federal de São PauloSão Paulo, Brazil; ^3^Department of Psychobiology, Universidade Federal de São PauloSão Paulo, Brazil; ^4^Faculty of Pharmaceutical Sciences, Universidade Estadual de CampinasCampinas, Brazil

**Keywords:** cocaine, drug abuse, mice, modafinil, open field, rapid-onset behavioral sensitization

## Abstract

There is substantial controversy about the addictive potential of modafinil, a wake-promoting drug used to treat narcolepsy, proposed as pharmacotherapy for cocaine abuse, and used indiscriminately by healthy individuals due to its positive effects on arousal and cognition. The rapid-onset type of behavioral sensitization (i.e., a type of sensitization that develops within a few hours from the drug priming administration) has been emerged as a valuable tool to study binge-like patterns of drug abuse and the neuroplastic changes that occur quickly after drug administration that ultimately lead to drug abuse. Our aim was to investigate the possible development of rapid-onset behavioral sensitization to modafinil and bidirectional rapid-onset cross-sensitization with cocaine in male Swiss mice. A priming injection of a high dose of modafinil (64 mg/kg) induced rapid-onset behavioral sensitization to challenge injections of modafinil at the doses of 16, 32, and 64 mg/kg, administered 4 h later. Furthermore, rapid-onset cross-sensitization was developed between modafinil and cocaine (64 mg/kg modafinil and 20 mg/kg cocaine), in a bidirectional way. These results were not due to residual levels of modafinil as the behavioral effects of the priming injection of modafinil were no longer present and modafinil plasma concentration was reduced at 4 h post-administration. Taken together, the present findings provide preclinical evidence that modafinil can be reinforcing *per se* and can enhance the reinforcing effects of stimulants like cocaine within hours after administration.

## Introduction

Most common drugs of abuse stimulate the release of dopamine in the mesoaccumbens dopaminergic system, which modulates both their reinforcing and psychomotor arousal effects (Wise and Bozarth, 1987; Alcaro et al., 2007). Within this context, it has been shown that the repeated administration of drugs of abuse promotes a progressive and long-lasting increase in the activity of the mesoaccumbens dopaminergic system, leading to a corresponding increase in their locomotor stimulatory effect in rodents (Vezina, 2004; Costa et al., 2007; O’Tuathaigh et al., 2010). This phenomenon, called behavioral sensitization, has been used to study the neurochemical mechanisms involved in the dopaminergic mesoaccumbens plasticity that are thought to play a major role in the reinforcing effects, incentive salience, and craving induced by drugs with abuse potential in humans (Robinson and Becker, 1986; Robinson and Berridge, 1993, 2008; Vezina et al., 2007). Notably, behavioral sensitization can be induced by short-term protocols. A challenge injection of cocaine (Valjent et al., 2010; Marinho et al., 2014), *d*-amphetamine (Frussa-Filho et al., 2004; Kameda et al., 2011), morphine (Vanderschuren et al., 2001; Marinho et al., 2015), or ethanol (Fukushiro et al., 2010) administered days, weeks or even months after a single drug injection can elicit behavioral sensitization. This single injection-induced locomotor sensitization protocol provides a useful model for investigating the long-lasting effects of drugs of abuse (Valjent et al., 2010).

Pioneering studies of Kuczenski and Segal (1999a,b) have also demonstrated that sensitization may develop in a very rapid manner. These authors showed that a few hours (3–5 h) after a priming injection of 4 mg/kg *d*-amphetamine, the administration of low, non-stereotypy-inducing doses of *d*-amphetamine (0.5–1.5 mg/kg) was able to elicit sensitization of stereotyped behaviors in rats. Later, evidence from our research group also showed the development of this rapid-onset sensitization phenomenon for the locomotor stimulant effect of *d*-amphetamine in mice (Alvarez et al., 2006; Chinen et al., 2006). Such finding is especially important within the context of “binge” patterns of stimulant abuse in humans, and suggests that neuroplastic events that mediate such abuse may occur in a rapid-onset manner.

An interesting feature of the behavioral sensitization phenomenon is the occurrence of cross-sensitization between different drugs of abuse such as *d*-amphetamine and cocaine (Suto et al., 2002), morphine and cocaine (McDaid et al., 2005), cocaine and ethanol (Manley and Little, 1997), and cocaine and nicotine (Collins and Izenwasser, 2004), suggesting common neurobiological mechanisms between these drugs.

Modafinil (diphenyl-methyl sulphonyl-2-acetamide) is a psychostimulant-like drug that acts as a wake-promoting substance and has been approved for the treatment of excessive daytime sleepiness in narcolepsy, obstructive sleep apnea and shift workers sleep disorder (Minzenberg and Carter, 2008). Modafinil also shows potential benefits for the treatment of psychiatric and neurologic disorders, including attention-deficit/hyperactivity disorder, cognitive deficits related to schizophrenia and Alzheirmer’s disease, sleepiness and fatigue related to Parkinson’s disease and amyotrophic lateral sclerosis (Ballon and Feifel, 2006; Minzenberg and Carter, 2008). Moreover, modafinil has been suggested as a reasonable medication for cocaine-addicted individuals (Dackis et al., 2003, 2005), with some studies showing that modafinil can attenuate the response to environmental cues related to cocaine use (Goudriaan et al., 2013), and reduce craving for cocaine (Hart et al., 2008). Although modafinil has emerged as a potential therapy for cocaine abuse, the safety of modafinil, with respect to its potential for abuse, has been questioned by other studies, mostly performed in animal models. For example, Gold and Balster (1996) showed the reinforcing and cocaine-like discriminative effects of modafinil in monkeys using the self-administration discrimination model.

It has been demonstrated that, similar to cocaine in humans, modafinil increases dopamine release in the nucleus accumbens by blocking the dopamine transporter (DAT) (Volkow et al., 2009; Funayama et al., 2014) and can produce withdrawal symptoms once its use is discontinued (Krishnan and Chary, 2015). In a study using animal models, Bernardi et al. (2009) have demonstrated that modafinil at high doses reinstated cocaine-induced conditioned place preference following extinction in rats. More recently, a study from our group demonstrated that modafinil exerts reinforcing effects, as it alone produces conditioned place preference and induces robust behavioral sensitization after single- and repeated-injection treatments in mice (Wuo-Silva et al., 2011). Although, sensitization induced by modafinil has been reported following repeated drug treatment or by acute administration of 64 mg/kg modafinil (Wuo-Silva et al., 2011), there is no evidence that modafinil can induce sensitization within hours of a single administration, in a protocol that could be compared to the binge pattern of drug abuse seen in humans. In addition, bidirectional cross-sensitization between modafinil and cocaine was also demonstrated in our previous study (Wuo-Silva et al., 2011). Even though there are several studies demonstrating the phenomenon of cross-sensitization between drugs, including modafinil and cocaine, using the classical sensitization model, there is none confirming that this phenomenon also occurs in the rapid-onset type of behavioral sensitization.

The present study aimed to investigate the possible development of rapid-onset behavioral sensitization to the locomotor-stimulating effect of modafinil and, subsequently, whether there would be a bidirectional rapid-onset cross-sensitization between modafinil and cocaine in mice.

## Materials and Methods

### Subjects

Male 3-month-old Swiss EPM-M2 mice (40–45 g) from our own colony were used. Animals were housed in polypropylene cages (33 cm × 44 cm × 17 cm) under conditions of controlled temperature (22–23°C) and lighting (12/12 h light/dark, lights on at 06:45 h). Food and water were available *ad libitum* throughout the experiments. Each cage contained animals from the same experimental group.

The experimental protocols were approved by the Committee for the Animal Care and Ethics of UNIFESP/SP [Universidade Federal de São Paulo (UNIFESP) #8030060514]. All animals were housed in a pathogen-free facility and were maintained in accordance with the National Institute of Health Guide for the Care and Use of Laboratory Animals (NIH Publications N° 8023), revised in 2011. All measures were taken to minimize the pain and discomfort of the animals.

### Drugs

Modafinil (16, 32, 64, and 80 mg/kg, CEPHALON^®^, Maisons-Alfort, France) was dissolved in 0.5% gum arabic and 0.9% NaCl (saline) solution. Cocaine-HCl (5, 10, and 20 mg/kg, Sigma-Aldrich, São Paulo, Brazil) was freshly diluted in 0.9% NaCl (saline) solution. Modafinil vehicle and saline were used as control solutions. The solutions were administered intraperitoneally (i.p.) at a volume of 10 ml/kg body weight.

### Behavioral Test: Open Field Test

Locomotor activity was measured in the open field, as previously described by Chinen et al. (2006). The open-field apparatus consisted of a circular wooden box (40 cm in diameter and 50 cm high) with an open top and a floor divided into 19 squares. Using hand-operated counters and stopwatches, the locomotion frequency (i.e., total number of entrances into any floor unit) was measured by an observer (who was blind to the treatment allocation) during a 10-min session. This interval has been proven effective in detecting modafinil and cocaine-induced behavioral sensitization induced by repeated treatment or a single injection in mice (Wuo-Silva et al., 2011; Marinho et al., 2015).

### Plasma Modafinil Concentrations

Blood samples were centrifuged at 3500 rpm for 10 min and plasma was extracted and immediately frozen at -80°C. Later, samples were thawed and plasma concentrations of modafinil were determined using liquid chromatography-tandem mass spectrometry (LC-MS/MS) conducted on a high performance liquid chromatography equipment Prominence system (Shimadzu, Kyoto, Japan). The analysis was conducted at the Forensic Toxicology Laboratory, Institute of Legal Medicine (Sao Paulo, Brazil).

### Experimental Procedures

#### Experiment 1. Rapid-Onset Behavioral Sensitization to the Locomotor Stimulating Effects of Modafinil

Eighty-four mice were habituated to the open field (10-min sessions) and to the injection procedure for three consecutive days, and their locomotor activity was measured on day 3. After the habituation phase, animals were allocated into seven groups of comparable basal locomotor activity (*n* = 12): Veh-Veh, Veh-Mod16, Mod64-Mod16, Veh-Mod32, Mod64-Mod32, Veh-Mod64, and Mod64-Mod64. On the 4th day, animals received an i.p. priming injection of vehicle solution (Veh-) or 64 mg/kg modafinil (Mod64-). Immediately after the injections, animals returned to their home cages. Four hours after their respective priming injections, animals received an i.p. challenge injection of vehicle (-Veh) or 16 mg/kg (-Mod16), 32 mg/kg (-Mod32), or 64 mg/kg (-Mod 64) modafinil. After 30 min, animals were placed individually in the open field and their locomotor activity was measured for 10 min.

The dose of modafinil for the priming injections was chosen based on a previous study from our laboratory demonstrating the development of robust behavioral sensitization induced by repeated administration of this dose of modafinil in mice (Wuo-Silva et al., 2011). The time interval between the priming injection and the challenge injection was based on previous studies by Kuczenski and Segal (1999a,b) and from our laboratory (Alvarez et al., 2006; Chinen et al., 2006) demonstrating the development of rapid-onset behavioral sensitization to *d*-amphetamine in rats and mice.

Experiments 2–4 were conducted in order to demonstrate that the changes on animals’ behavior observed in Experiment 1 were not due to residual levels of the priming injection of modafinil at the moment of the challenge session.

#### Experiment 2. Time-Response Curve of the Locomotor Stimulating Effect Induced by 64 mg/kg Modafinil Acute Administration in Mice

Twenty mice were habituated to the open field (10-min sessions) and to the injection procedure for three consecutive days, and their locomotor activity was measured on day 3. After the habituation phase, animals were allocated into two groups of comparable basal locomotor activity (*n* = 10): Veh and Mod64. On the 4th day, animals received either an i.p. injection of vehicle solution (Veh) or 64 mg/kg modafinil (Mod64). Thirty min later, locomotor activity was measured for 10 min, every 30 min, for a total period of 240 min.

#### Experiment 3. Plasma Concentrations of Modafinil 30 min and 4 h after Acute Administration of 64 mg/kg Modafinil

Eight mice were allocated into two groups (*n* = 4): Mod30min and Mod4h. Animals from both groups received an i.p. injection of 64 mg/kg modafinil at the same time. One group of mice was euthanized 30 min after administration of modafinil (Mod30min group), and the other group 4 h later (Mod4h group). Animals were euthanized by decapitation and blood was collected in microtubes for subsequent quantification of modafinil concentrations in plasma.

#### Experiment 4. Effects of a Residual Dose of Modafinil on the Locomotor Stimulating Effect of 64 mg/kg Modafinil

To verify whether the residual levels of modafinil found in the plasma of animals 4 h after drug administration would affect locomotor activity of mice, we performed Experiment 4. For this purpose, we compared the effects of 64 mg/kg modafinil (highest dose of modafinil used during the challenge session of Experiment 1) with the effects of 80 mg/kg modafinil on locomotor activity of separate groups of animals. This higher dose of modafinil was calculated by combining the 64 mg/kg dose of the challenge session with the residual levels of modafinil found in the plasma of animals (16 mg/kg). To find out the residual dose in mg/kg to be administered in combination with the 64 mg/kg dose of modafinil, we converted mg/kg to the same unit of the plasma concentrations (mg/ml) and considered the first plasma measurement at 30 min (66.5 mg/ml) equivalent to the dose that was administered to mice (64 mg/kg = 6.4 mg/ml).

Twenty-nine mice were habituated to the open field (10-min sessions) and to the injection procedure for three consecutive days, and their locomotor activity was measured on day 3. After the habituation phase, animals were allocated into three groups of comparable basal locomotor activity: Veh (*n* = 10), Mod64 (*n* = 9), and Mod80 (*n* = 10). On the 4th day, animals received an i.p. injection of vehicle solution (Veh), 64 mg/kg modafinil (Mod64) or 80 mg/kg modafinil (Mod80). After 30 min, animals were individually placed in the open field and the locomotor activity was measured for 10 min.

#### Experiment 5. Rapid-Onset Behavioral Cross-Sensitization to Cocaine after a Priming Injection of Modafinil

Eighty-four mice were habituated to the open field (10-min sessions) and to the injection procedure for three consecutive days, and their locomotor activity was measured on day 3. After the habituation phase, animals were allocated into seven groups of comparable basal locomotor activity (*n* = 12): Veh-Sal, Veh-Coc5, Mod64-Coc5, Veh-Coc10, Mod64-Coc10, Veh-Coc20, and Mod64-Coc20. On the 4th day, animals received an i.p. priming injection of vehicle solution (Veh-) or 64 mg/kg modafinil (Mod64-). Immediately after the injections, animals returned to their home cages. Four hours after their respective priming injections, animals received an i.p. challenge injection of saline solution (-Sal) or 5 mg/kg (-Coc5), 10 mg/kg (-Coc10), or 20 mg/kg (-Coc20) cocaine. After 5 min, animals were individually placed in the open field and the locomotor activity was measured for 10 min.

#### Experiment 6. Rapid-Onset Behavioral Cross-Sensitization to Modafinil after a Priming Injection of Cocaine

Eighty-four mice were habituated to the open field (10-min sessions) and to the injection procedure for three consecutive days, and their locomotor activity was measured on day 3. After the habituation phase, animals were allocated into seven groups of comparable basal locomotor activity (*n* = 12): Sal-Veh, Sal-Mod16, Coc20-Mod16, Sal-Mod32, Coc20-Mod32, Sal-Mod64, and Coc20-Mod64. On the 4th day, animals received an i.p. priming injection of saline solution (Sal-) or 20 mg/kg cocaine (Coc20-). Immediately after the injections, animals returned to their home cages. Four hours after their respective priming injections, animals received an i.p. challenge injection of vehicle solution (-Veh) or 16 mg/kg (-Mod16), 32 mg/kg (-Mod32), or 64 mg/kg (-Mod64) modafinil. After 30 min, animals were individually placed in the open field and the locomotor activity was measured for 10 min.

The doses of cocaine for Experiments 5 and 6 were chosen based on a previous study showing time-effect curves of the locomotor stimulant effect induced by acute cocaine administration in mice (Gatch et al., 2013).

## Results

### Experiment 1. A Priming Injection of Modafinil Induced Rapid-Onset Behavioral Sensitization to a Challenge Injection of Modafinil at Several Doses

**FIGURE 1:**
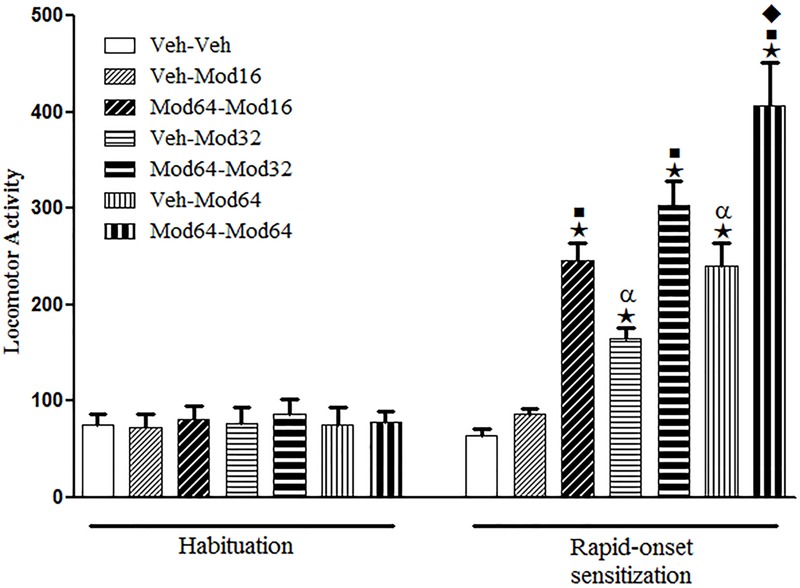
**Locomotor activity during the 3rd day of habituation and during the challenge session of the rapid-onset behavioral sensitization test.** The animals received a priming injection of vehicle (Veh-) or 64 mg/kg modafinil (Mod64-) and, after 4 h, a challenge injection of vehicle (-Veh) or 16 mg/kg (-Mod16), 32 mg/kg (-Mod32), or 64 mg/kg modafinil (-Mod64). After 30 min, the locomotor activity was measured for 10 min in the open field. Data are reported as mean ± SEM (*n* = 12). ★*P* < 0.05 compared with the Veh-Veh group. ■*P* < 0.05 compared with the respective control group, which received a priming injection of vehicle. α*P* < 0.05 compared with the Veh-Mod16 group. ◆*P* < 0.05 compared with all of the other groups.

Regarding the sensitization test (day 4), one-way ANOVA revealed significant group differences [*F*(6,77) = 27.2, *P* < 0.05]. Tukey’s *post hoc* test revealed that with respect to animals acutely treated with modafinil for the first time (priming injection of vehicle and challenge injection of modafinil), only the Veh-Mod32 and Veh-Mod64 groups presented a significant increase in locomotion when compared with the Veh-Veh control group, indicating that only the highest doses of modafinil were effective in promoting locomotor stimulant effects. Furthermore, the statistical analysis revealed that animals treated with a priming injection of 64 mg/kg modafinil and challenged with different doses of modafinil (Mod64-Mod16, Mod64-Mod32, and Mod64-Mod64 groups) presented significantly greater locomotor activity when compared to their respective control groups treated initially with vehicle and challenged with different doses of modafinil (Veh-Mod16, Veh-Mod32, and Veh-Mod64 groups), characterizing the development of rapid-onset behavioral sensitization to modafinil for all doses. In addition, the magnitude of rapid-onset sensitization was greater for the highest dose of modafinil during challenge session, as animals of the Mod64-Mod64 group presented significantly greater locomotor activity than animals of the Mod64-Mod16 and Mod64-Mod32 groups (**Figure 1**).

### Experiments 2–4. Behavioral Effects and Modafinil Plasma Concentrations 4 h after 64 mg/kg Modafinil Acute Administration

**FIGURE 2:**
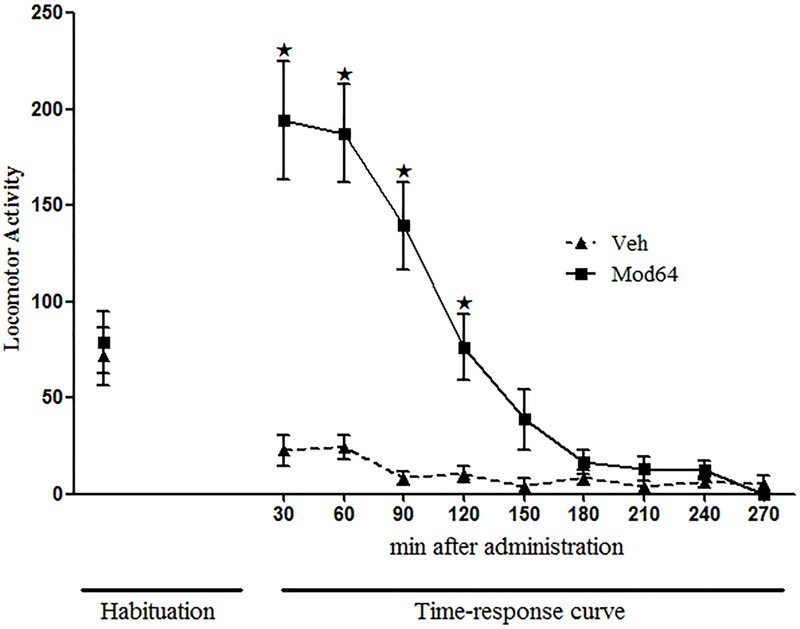
**Locomotor activity in the open field during the 3rd day of habituation and throughout 270 min after acute administration of vehicle (Veh) or 64 mg/kg modafinil (Mod64).** During the open-field test, locomotor activity was measured for 10 min, every 30 min, for a period of 240 min. Data are reported as mean ± SEM (*n* = 10). ★*P* < 0.05 compared with Veh group at the respective time interval.

**Figure 2** shows the locomotor activity of animals on the 3rd day of habituation and during 240 min after acute administration of 64 mg/kg modafinil. Two-way ANOVA revealed significant effects of time (minutes of observation) [*F*(1,18) = 31.3, *P* < 0.05], treatment (Sal × Mod64) [*F*(1,18) = 33.5, *P* < 0.05] and a significant time × treatment interaction [*F*(1,18) = 22.0, *P* < 0.05]. Tukey’s *post hoc* test revealed that animals that received 64 mg/kg modafinil had a significantly higher locomotor activity compared to animals of the Veh group at 30, 60, 90, and 120 min post-injection. After 120 min, animals of the Mod64 group showed no significant differences in locomotor activity relative to animals from the Veh group. These results show that after 4 h, there was no longer a stimulating effect of modafinil on animals’ locomotion.

The plasma analysis showed that there was a significant decrease in the concentration of modafinil in blood samples taken 4 h after modafinil (17.3 ± 3.13 mg/ml) administration (Mod4h group) compared to blood samples taken 30 min (66.5 ± 3.03 mg/ml) after administration (Mod30min group) [Student’s *t*-test: *t*(6) = 11.3, *P* < 0.0001].

**FIGURE 3:**
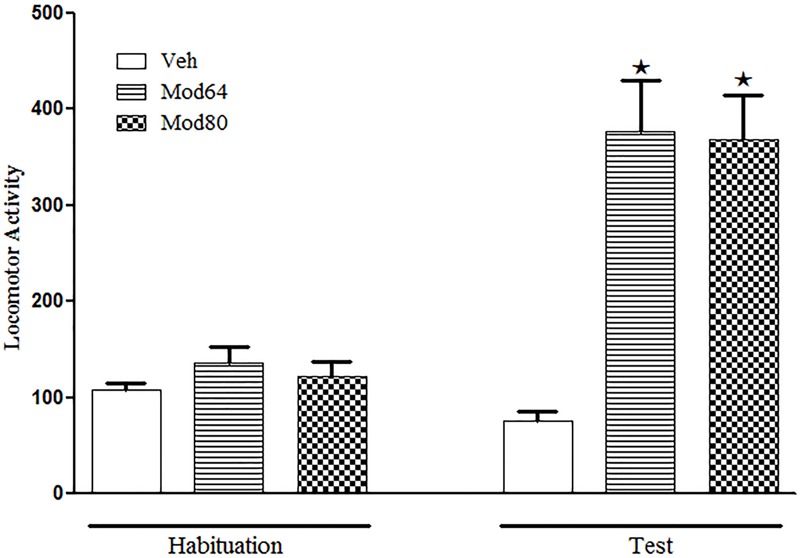
**Locomotor activity in the open field during the 3rd day of habituation and during the test session with vehicle or two different doses of modafinil.** The animals received an injection of vehicle solution (Veh), 64 mg/kg (Mod64) or 80 mg/kg (Mod80) modafinil, and after 30 min, the locomotor activity was measured for 10 min in the open field. Data are reported as mean ± SEM. Veh (*n* = 10), Mod64 (*n* = 9), and Mod80 (*n* = 10). ★*P* < 0.05 compared with the Veh group.

With respect to Experiment 4, one-way ANOVA revealed significant differences between groups on the test day (day 4) [*F*(2,26 = 18.5, *P* < 0.05]. Tukey’s *post hoc* test revealed that the animals treated acutely with 64 or 80 mg/kg modafinil (Mod64 and Mod80 groups) presented a significant increase in locomotion when compared to the Veh group, demonstrating the locomotor stimulant effect of both doses of modafinil. Importantly, the statistical analysis showed no significant differences in locomotion of the animals treated with 64 mg/kg modafinil compared to animals treated with 80 mg/kg modafinil (**Figure 3**).

### Experiment 5. A Priming Injection of Modafinil Induced Rapid-Onset Cross-Sensitization with a Challenge Injection of Cocaine

**FIGURE 4:**
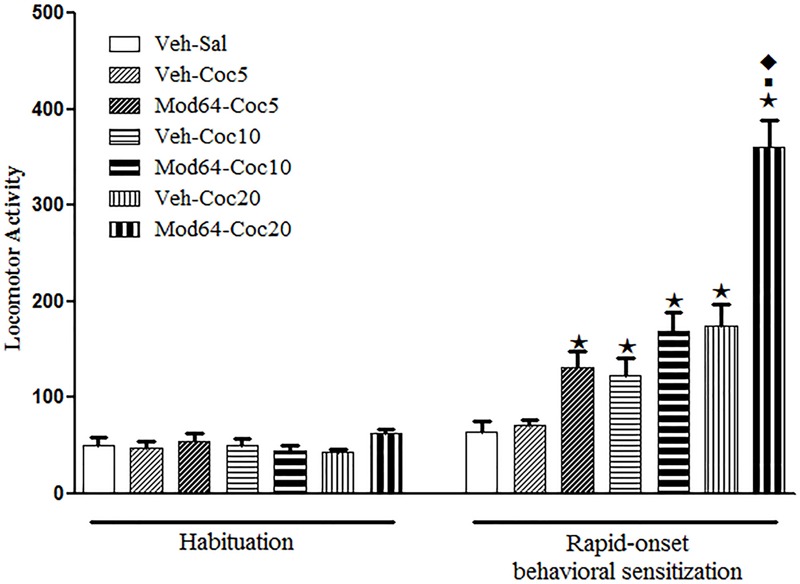
**Locomotor activity during the 3rd day of habituation and during the challenge session of the rapid-onset behavioral sensitization test with saline or different doses of cocaine.** The animals received a priming injection of modafinil vehicle solution (Veh-) or 64 mg/kg modafinil (Mod64-), and after 4 h, a challenge injection of saline (-Sal), 5 mg/kg (-Coc5), 10 mg/kg (Coc10), or 20 mg/kg (-Coc20) cocaine. After 5 min, the locomotor activity was measured for 10 min in the open field. Data are reported as mean ± SEM (*n* = 12). ★*P* < 0.05 compared with the Veh-Sal group. ■*P* < 0.05 compared with the respective control group that received a priming injection of vehicle solution. ◆*P* < 0.05 compared with all of the other groups.

Concerning the sensitization test (day 4), one-way ANOVA revealed significant differences between groups [*F*(6,77) = 28.6, *P* < 0.05]. Tukey’s *post hoc* test revealed that only the groups treated acutely with high doses of cocaine (Veh-Coc10 and Veh-Coc20) presented a significant increase in locomotion when compared to the Veh-Sal group, which demonstrated the locomotor stimulant effect of cocaine at these doses. Moreover, the locomotor effects of a challenge injection of 20 mg/kg cocaine were enhanced in mice pre-exposed to 64 mg/kg modafinil (Mod64-Coc20), as compared to mice pre-exposed to vehicle solution (Veh-Coc20), revealing a rapid-onset cross-sensitization between modafinil and a high dose of cocaine. Indeed, the locomotor activity presented by the Mod64-Coc20 group was significantly greater than all of the others groups (**Figure 4**).

### Experiment 6. A Priming Injection of Cocaine Induced Rapid-Onset Cross-Sensitization with a Challenge Injection of Modafinil

**FIGURE 5:**
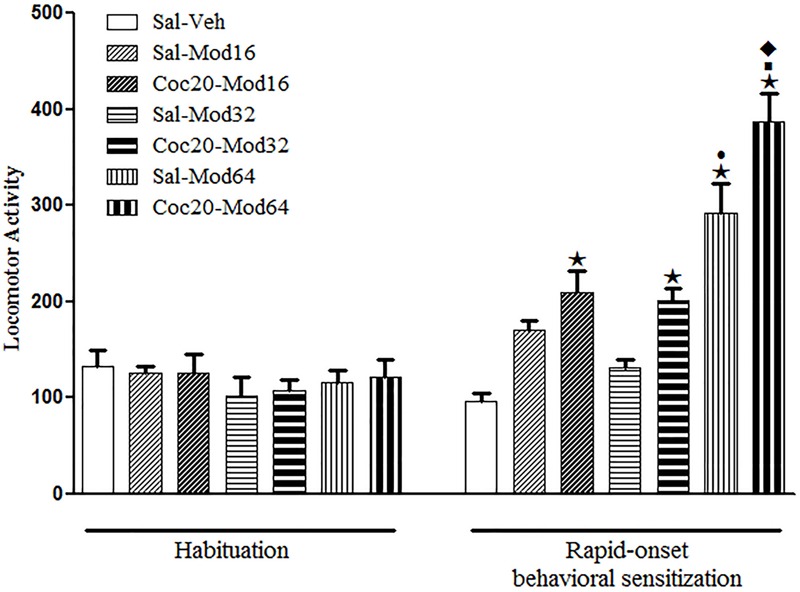
**Locomotor activity during the 3rd day of habituation and during the challenge session of the rapid-onset behavioral sensitization test with vehicle or different doses of modafinil.** The animals received a priming injection of saline (Sal-) or 20 mg/kg cocaine (Coc20-), and after 4 h, a challenge injection of modafinil vehicle solution (-Veh), 16 mg/kg (-Mod16), 32 mg/kg (-Mod32), or 64 mg/kg (-Mod64) modafinil. After 30 min, the locomotor activity was measured for 10 min in the open field. Data are reported as mean ± SEM (*n* = 12). ★*P* < 0.05 compared with the Sal-Veh group. ■*P* < 0.05 compared with the respective control group that received a priming injection of saline. •*P* < 0.05 compared with the Sal-Mod16 and Sal-Mod32 groups. ◆*P* < 0.05 compared with all of the other groups.

During the sensitization test, significant differences between groups were detected by one-way ANOVA [*F*(6,77) = 24.9, *P* < 0.05]. Tukey’s *post hoc* test revealed that among the groups previously treated with saline, only the Sal-Mod64 group, treated acutely with the highest dose of modafinil, had increased locomotor activity when compared to the Sal-Veh group, which demonstrated that only this dose was effective in inducing a locomotor stimulant effect in mice. Importantly, the locomotor effects of a challenge injection of 64 mg/kg modafinil were enhanced in mice previously treated with 20 mg/kg cocaine (Coc20-Mod64 group), as compared to mice previously treated with saline (Sal-Mod64), revealing a rapid-onset cross-sensitization between cocaine and a high dose of modafinil (**Figure 5**).

## Discussion

The present study shows the following main findings: (1) a priming injection of a high dose of modafinil (64 mg/kg) induced rapid-onset sensitization to the locomotor stimulant effects of low and high doses of modafinil administered 4 h later; and (2) there was bidirectional rapid-onset cross-sensitization between modafinil and cocaine, i.e., a modafinil priming injection induced rapid-onset behavioral sensitization to a subsequent cocaine challenge injection, and a cocaine priming injection induced rapid-onset behavioral sensitization to a modafinil challenge injection.

This is the first paper showing that behavioral sensitization to modafinil can develop in such a rapid manner. As previously mentioned, it has been demonstrated that a priming injection of *d*-amphetamine at a high dose can elicit intense stereotyped behaviors and high levels of locomotor activity in rats and mice challenged with a sub-threshold dose of *d*-amphetamine a few hours later (3–5 h) (Kuczenski and Segal, 1999a,b; Alvarez et al., 2006; Chinen et al., 2006). The present study adds to the literature by showing that modafinil was also able to produce this rapid-onset type of behavioral sensitization in mice. Behavioral sensitization induced by repeated treatment with a drug in rodents has been extensively associated with increased activity in the mesoaccumbens dopaminergic system (Robinson and Berridge, 2008). As for the rapid-onset behavioral sensitization, the only data available regarding the underlying mechanisms are those reported by Kuczenski and Segal (1999b), which demonstrated that both dopamine D1 and D2 receptors are important for the induction and expression of rapid-onset sensitization of stereotyped behaviors induced by *d*-amphetamine in rats. Thus, the demonstration that modafinil effectively produces rapid-onset locomotor sensitization provides further support for the mesolimbic dopaminergic mechanism of modafinil and suggests that a single dose of modafinil can immediately lead to neuroplastic mechanisms thought to be involved in drug abuse.

In fact, such neuroplastic changes can occur immediately after the administration of a drug of abuse. In a recent study, Muñoz-Cuevas et al. (2013) demonstrated *in vivo* the emergence of new dendritic spines in the dorsomedial prefrontal cortex of mice only 2 h after administration of cocaine that correlated positively with cocaine-induced effects on animals’ behavior. In addition, Kuczenski and Segal (1999a) demonstrated that sub-threshold doses of both dopamine D1 and D2 receptors agonists promoted stereotyped behaviors in rats primed with an amphetamine injection a few hours before, suggesting that this single injection of amphetamine enhanced sensitivity of both D1 and D2 receptors in a very short time frame. The present data, together with previous ones from our research group (Wuo-Silva et al., 2011), reinforce the hypothesis that modafinil alone has significant reinforcing effects that should not be ruled out.

Our results are contrary to the studies of Deroche-Gamonet et al. (2002) and Shuman et al. (2012), which suggest that modafinil would be devoid of abuse potential. These studies showed that modafinil was not able to induce conditioned place preference, self-administration, or behavioral sensitization in rodents, indicating that modafinil would not have reinforcing properties. However, there are important methodological differences between those studies and ours that could explain this discrepancy in results. In our study, mice were placed in the open-field apparatus 30 min after modafinil administration, where they remained for 10 min, while Deroche-Gamonet et al. (2002) administered modafinil and immediately placed the animals in the test apparatus. It is likely that because of this lack of time between administration of modafinil and the introduction of animals into the apparatus the behavioral effects induced by modafinil were not observed at its peak effect. With respect to the absence of modafinil-induced behavioral sensitization in mice reported by Shuman et al. (2012), it is worth noting that these authors evaluated the effects of modafinil on the sensitization model following repeated administration of the drug (classical sensitization protocol) and by challenging the animals with a very low dose of modafinil (0.75 mg/kg). In addition, sensitization was measured in the conditioned place preference apparatus, which is most commonly used to measure the reinforcing properties of drugs but not exactly the best apparatus to measure the effects of drugs on locomotor activity of rodents. Notwithstanding, Shuman et al. (2012) did observe modafinil-induced conditioned place preference and an interaction of modafinil and cocaine in the classical behavioral sensitization model, similar to what we reported in our previous study (Wuo-Silva et al., 2011) and what was reported in the study from Nguyen et al. (2011).

One could argue that the behavioral results presented here could be due to residual levels of the priming injection of modafinil at the moment of the challenge session. However, this possibility seems unlikely for several reasons. First, Experiment 2 demonstrated that 150 min after an acute injection of modafinil at the same dose as the priming injection for Experiments 1 and 5 (64 mg/kg), the locomotor stimulating effect had ceased completely. These results corroborate the findings of Duteil et al. (1990), which showed that the locomotor stimulant effect of 64 mg/kg modafinil persisted for a period of 120 min in mice. Second and supporting the behavioral data, blood sample analysis (Experiment 3) indicated that modafinil plasma levels decreased significantly 4 h after drug administration. Additionally, it is worth mentioning that microdialysis studies in rats (Zolkowska et al., 2009) and rhesus monkeys (Andersen et al., 2010) showed that extracellular dopamine levels have a peak at 20 min after administration of high doses of modafinil, returning to baseline levels up to 100 min later. However, Loland et al. (2012) reported that dopamine levels remained elevated in the nucleus accumbens shell of mice for 6 h after modafinil administration. The results presented by these authors are not compatible with the results found in our study for the measurements of modafinil in plasma of mice. Of note, Loland et al. (2012) used different doses of modafinil (30, 100, and 300 mg/kg) than that used in our study (64 mg/kg). This difference might have influenced the somewhat discrepant findings between the studies. We should also point out that the lack of measurement of brain levels of modafinil is a limitation of our study. Studies have shown that cocaine concentration in the brain appears to be more reliable than plasma drug levels to predict behavioral changes in rodents (Reith et al., 1987; Zombeck et al., 2009). However, there are no studies showing a potential correlation between modafinil levels in the brain and locomotor activity in rodents. Studies have only assessed the levels of dopamine and its metabolites in the brain following modafinil administration (see Andersen et al., 2010; Mereu et al., 2016). Hereafter, further studies can be performed in order to associate modafinil levels in the brain and behavioral responses in mice, thereby providing important insights into the mechanisms of the rapid-onset behavioral sensitization phenomenon induced by modafinil.

Despite the significant reduction in modafinil plasma levels described in Experiment 3, considerable levels of modafinil in the plasma of animals were still detected 4 h after drug administration. One could still speculate that this plasma residue of modafinil might be able to interfere with the behavior of animals and contribute to increase their locomotor activity during the challenge session of the sensitization test. However, this does not seem to be the case, as Experiment 4 demonstrated that the animals receiving the dose of 80 mg/kg modafinil (calculated residual dose combined with the challenge dose) showed no significant difference in locomotor activity when compared to mice receiving only the challenge dose of 64 mg/kg modafinil. Another point raised here is that the lack of difference in the locomotor stimulant effect induced by 64 mg/kg modafinil compared to 80 mg/kg modafinil could be due to a ceiling effect. This possibility seems unlikely, as we have previously shown that the acute effect of a very high dose of modafinil (128 mg/kg) was significantly greater than the locomotor-activating effect of 64 mg/kg modafinil in the same mouse strain (Wuo-Silva et al., 2011). In addition, Duteil et al. (1990) have shown similar results, i.e., dose-dependent increases in locomotor activity of mice induced by the doses of 32, 64, and 128 mg/kg modafinil.

Altogether, data from Experiments 2–4 support our hypothesis that the enhanced locomotion observed in mice receiving two modafinil injections at a 4-h interval demonstrates the expression of a rapid-onset behavioral sensitization and is not due to residual levels of the drug.

Supporting the idea that modafinil shares common plastic neuronal mechanisms related to drug abuse, Experiments 5 and 6 demonstrated a bidirectional rapid-onset cross-sensitization between modafinil and cocaine. In fact, there was an enhancement of the locomotor stimulant effect of a challenge injection of 20 mg/kg cocaine 4 h after administration of 64 mg/kg modafinil, as well as an enhancement of the locomotor stimulant effect of a challenge injection of 64 mg/kg modafinil 4 h after administration of 20 mg/kg cocaine. These results are in agreement with our previous study (Wuo-Silva et al., 2011) showing that pre-treatment with repeated injections of cocaine enhanced the locomotor stimulant effects of an acute injection of modafinil, and pre-treatment with repeated injections of modafinil enhanced the locomotor stimulant effects of an acute injection of cocaine in mice. Similarly, Soeiro et al. (2012) demonstrated cross-sensitization between methamphetamine and modafinil in mice. Altogether, the bidirectional cross-sensitization between modafinil and other psychostimulants seen in the present study and in previous ones suggests that modafinil shares common neurobiological mechanisms with psychostimulants related to both the induction and the expression phases of sensitization. Within this context, while events in the ventral tegmental area have been linked to the induction of sensitization, events in the nucleus accumbens seem to be responsible for the expression of this phenomenon (Pierce and Kalivas, 1997).

Recent studies have shown that modafinil can also be reinforcing to humans, as it can increase incentive salience (Smart et al., 2013), motivation for reinforcement seeking (Young and Geyer, 2010) and reward anticipation (Funayama et al., 2014). Evidence suggests that the reinforcing effects of modafinil may be related to the dopaminergic system, as seen for most of the drugs with abuse potential. It has been shown that modafinil increased dopamine levels in the nucleus accumbens shell and core of mice (Mereu et al., 2016) at levels similar to those induced by typical psychostimulants, and that this enhancement was due to blockade of DAT (see Volkow et al., 2009; Andersen et al., 2010; Kim et al., 2014). Zolkowska et al. (2009) demonstrated that modafinil-induced increases in locomotor activity of rats are mediated by this activity of modafinil on DAT and subsequent increases on nucleus accumbens dopamine levels. Similarly, Andersen et al. (2010) demonstrated that modafinil reinstated cocaine-seeking behavior in rhesus monkeys by blocking DAT and increasing extracellular dopamine concentrations in the caudate-putamen.

Our results are also clinically relevant as they raise concerns about the prescription of modafinil for drug abuse treatment (Dackis et al., 2003, 2005, 2012; Hart et al., 2008). Hart et al. (2008) and Vosburg et al. (2010) reported that modafinil reduced measurements of subjective effects in cocaine addicts, such as craving and reinforcement. However, it is important to emphasize that even clinical studies that propose the use of modafinil for the treatment of drug of abuse are contradictory. For example, Dackis et al. (2005) demonstrated that modafinil decreased cocaine levels in urine samples of cocaine addicts. In contrast, in a more recent study, Dackis et al. (2012) showed that modafinil failed to decrease the positive urine samples for cocaine in drug addicts. Another concern regarding the clinical relevance of our study is the increasing use of modafinil for non-medical purposes, such as to increase cognitive ability (Cakic, 2009), to sustain alertness in military pilots (Estrada et al., 2012) and even to improve performance in athletes (Strano Rossi and Botrè, 2011). Of note, recent studies have demonstrated that modafinil produces a number of serious side effects, such as psychotic symptoms (Wu et al., 2008) and severe cutaneous (Stevens-Johnson Syndrome) and cardiovascular adverse reactions (Carstairs et al., 2010). These studies, along with other data in the literature, led the European Medicines Agency and the Agency’s Committee for Medicinal Products for Human Use (2011) to restrict the use of modafinil only for the treatment of narcolepsy. Given the conflicting data regarding the effects of modafinil and its abuse liability demonstrated in the present study, it could be expected that, in the near future, modafinil might be classified as a drug of abuse.

## Author Contributions

RW-S, DFF, RF-F, and BL were responsible for the study concept and design. RW-S, DFF, AH, RS-B, EM-K, LB, TY, LL-S, CB, RP-S, DH, LF, and JC contributed to the acquisition of animal data. RW-S, DFF, RF-F, and BL assisted with data analysis and interpretation of findings. RW-S, DFF, and BL drafted the manuscript. All authors critically reviewed content and approved the final version for publication.

## Conflict of Interest Statement

The authors declare that the research was conducted in the absence of any commercial or financial relationships that could be construed as a potential conflict of interest.
